# The Effect of Perturbation-Based Balance Training vs Step Training on Reaction Time in Older Persons: A Review

**DOI:** 10.7759/cureus.48104

**Published:** 2023-11-01

**Authors:** Anushka P Bhagwat, Nishigandha P Deodhe

**Affiliations:** 1 Department of Neurophysiotherapy, Ravi Nair Physiotherapy College, Datta Meghe Institute of Higher Education and Research, Wardha, IND

**Keywords:** perturbation-based balance, older persons, perturbation, balance training, reaction time, older adults, step training

## Abstract

As the global population ages, understanding the impact of aging on cognitive abilities becomes increasingly crucial. One such cognitive function that exhibits significant changes with advancing age is reaction time (RT). This article presents a comprehensive analysis of RT in older adults, examining its underlying mechanisms, age-related changes, and implications for cognitive functioning. Through a review of existing literature, this study explores various factors influencing RT in older individuals, including sensory, motor, and cognitive processes. It highlights how age-related changes in the central nervous system, such as declines in processing speed and neural efficiency, play a significant role in prolonging RTs. Additionally, the role of cumulative life experiences and environmental factors in shaping reaction time in older adults is discussed.

## Introduction and background

In behavioral sciences, reaction time (RT) is a typical trait that is regarded to be a valuable measure of how rapidly and efficiently brain processes are operating. Despite its widespread use, there are a number of disadvantages to RT, particularly in differential and developmental research [1]. The slow and ongoing aging of the world's population is having a profound impact on the global demographic landscape. This change in the population is a result of better healthcare, higher living standards, and longer life spans. Senior individuals have a variety of physiological changes as they age; however, these changes can have a significant impact on their general well-being. A decrease in balance is one such physiological condition that occurs more frequently as we age. The loss of balance, which is frequently accompanied by an increased risk of falling, can significantly affect how older people live their everyday lives. Given the possibility of catastrophic injury and death, the increased risk of falling for the elderly can be a significant reason for concern. In this setting, exercise stands out as a potent and scientifically supported technique to stave against balance loss in older people. Even as people age, regular physical activity and exercise improve balance, stability, and all other aspects of physical function. From low-impact exercises like tai chi and yoga to more demanding strength training and cardiovascular workouts, these activities cover a wide variety of possibilities. Such activities not only support and enhance physical balance but also advance cardiovascular health, muscle strength, and flexibility. It is impossible to stress the importance of exercise as a safeguard against balance loss. In addition to lowering the risk of falls, it also improves the quality of life for the elderly. Physical exercise gives older adults the tools they need to live more independent and meaningful lives, enabling them to take full advantage of their golden years. This is done by encouraging active and healthy aging. As a result, comprehensive healthcare and support systems created to suit the particular requirements of this expanding group must include encouraging and enabling physical exercise among the elderly [2]. Critical information regarding cognitive and emotional processes, as well as RT, may be learned from a response's intensity. The experiment measuring response force in a simple reaction task to visual stimuli of increasing brightness and size is used to assess the potential of the RT [3]. However, as we age, our RT gradually lowers. The primary reason is decreased or impaired cognitive performance. A decrease in basic RT is directly linked to falls in elderly adults. This is especially true for activities that require multitasking, heightened motor responsiveness, and sophisticated thought [4]. Fall is the leading cause of injury for those over the age of 65, which is a severe problem for the elderly. The complicated biological process of maintaining balance involves the cerebral cortex processing sensory inputs from the vestibular, visual, and proprioceptive systems, as well as posture and movement control via the cerebellum [5]. Although the causes of falling are diverse, the capacity to properly adapt to a quick, unanticipated balance disturbance (either "external," such as slips or trips, or "internal," owing to self-initiated movement) ultimately decides whether a fall happens [6]. Balance recovery reflexes involving quick stepping or gripping motions are especially important [7]. These support-change reactions are the only defense against significant balance disturbances and are widespread responses to minor perturbations [8]. The primary characteristic of any balancing disturbance is that it causes relative displacement between the center of mass (COM) and the base of support (BOS) [9]. Stepping is a frequently used defensive tactic for preserving equilibrium in daily life, according to a growing body of research [5-10]. Steps can be taken proactively to prevent falls or reactively in response to balance problems from the outside world. In general, older persons, particularly those with a history of falls, are more likely than younger ones to take precautionary steps to maintain their balance. As a result, it is critical for the elderly to be skilled at using safe stepping practices [10-12]. As a result, it is vital for elderly individuals to be effective in implementing protective stepping measures. Task-specific training can increase RT performance in both healthy and diseased populations [13,14]. As a result, it is possible that RT in voluntary stepping may improve. It may be informative to compare the training effects of voluntary stepping practice vs perturbation-induced stepping [10].

## Review

Data sources and search engine 

This review included studies from original papers, systematic reviews, meta-analyses, and randomized controlled trials (RCTs). The papers were found using keywords and Medical Subject Headings (MESH) phrases. Keywords were also used to screen the articles. For selecting the articles, the keywords used were reaction time, reduced risk of fall, balance training, and step training. The online search engines PubMed, Scopus, Web of Science, and Google Scholar were used to collect articles.

Methodology

After removing 12 unrelated studies and duplicates, a total of 21 records of systematic literature were appraised for eligibility as full-text publications. This review includes a total of seven studies, which are listed below. The inability to access the complete version of articles was one of the primary grounds for exclusion. As a result, additional literature on the subject is required. 

Eligibility Criteria

Inclusion criteria: Age > 60 years, balance impairment (BBS score of 0-20 as it signals balance impairment), walking ability: should be able to walk freely with or without support.

Exclusion criteria: History of injuries, individuals having balance impairment due to higher mental disorder, participants not having any recent history of lower limb injury, and patient having neuropathy.

Search strategy

**Figure 1:**
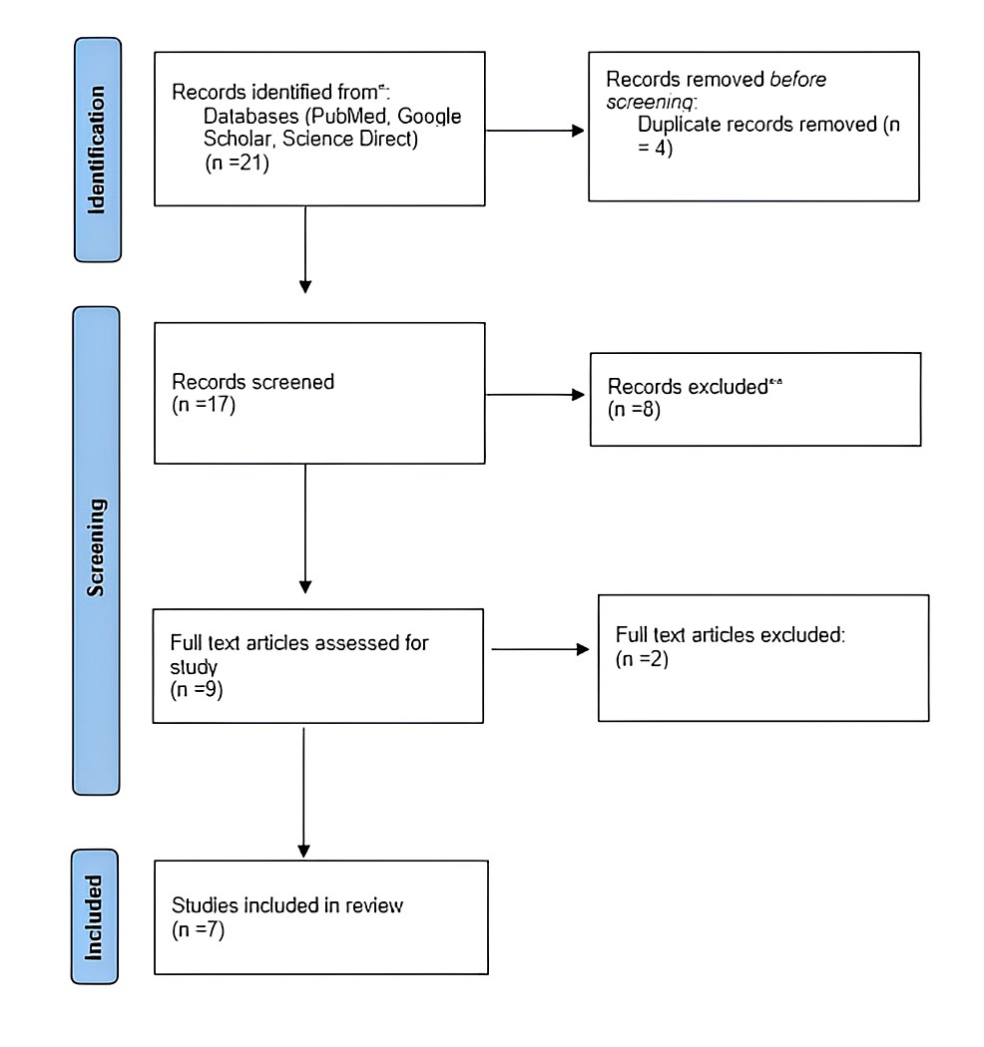
An overview of the publications

There were several research types, such as experimental investigations, RCTs, systematic reviews, and literature reviews. Figure 1 provides an overview of the publications chosen in accordance with Preferred Reporting Items for Systematic Reviews and Meta-Analyses (PRISMA) recommendations. 

Reaction time in older adults

It is well established that fundamental sensory stimuli (such as light flashes or auditory stimuli) become more intense with age [15-17]. Although the effects of aging on responses to increasingly complex visual stimuli are less well understood, it is unclear whether this is due to an increase in sensory or motor components. In many circumstances in everyday life, responding to moving stimuli is crucial. Older individuals may be at risk for danger if their RTs to moving stimuli rise significantly [18]. It was demonstrated in a prior research study (Burr, Fiorentini, & Morrone, 1998) that the perceived stimulus velocity, rather than the actual stimulus velocity, determines the RT to the grating motion beginning for both pure luminance and pure color contrast gratings (see also Ball & Sekuler, 1980) [19,20].

Perturbation-based balance training and step training

Perturbation-based balance training (PBT) is defined as balance training that involves repetitive, externally delivered mechanical perturbations to induce quick reactions in a safe and controlled setting to restore postural stability. PBT aims to precisely target and increase the capacity to regain equilibrium in destabilizing conditions such as those that contribute to falls in everyday life. To be considered PBT, the training must satisfy two fundamental characteristics [1]. External perturbations that cause a sudden motor reaction should be used in the training [2]. These disruptions should be significant enough to produce a loss of stability, resulting in a fall without a sufficient motor response (or use of the safety harness). A biomechanical loss of stability occurs when the position and motion characteristics of the center of mass exceed specific spatial and temporal limitations relative to the BOS, signaling that a fall is imminent if no extra action is done [21,22]. PBT-induced motor adjustments might be either predictive or reactive in character [23,24]. In practice, this rapid reaction to a loss of stability when standing or walking typically manifests as reactive stepping or reaching (where appropriate support is available), as defined by Maki and McIlroy as a change-in-support strategy [25]. Gait stability adaptations and fall risk reduction have both been achieved through the use of PBT. Specific brain structures must be activated in order for compensatory reactions to be produced in response to environmental perturbations. This implies that training balance recovery reactions ought to demonstrate acute training impacts on cognition [21].

Stepping is a typical protective mechanism for maintaining balance in the everyday world, according to a growing body of research [10,26-28]. Steps can be taken voluntarily to prevent a fall or reactively in response to external challenges to balance. In general, older people, particularly those with a history of falls, are more likely than younger people to adopt precautionary measures to preserve their balance [11,29]. Step training with preparation signals can improve stability limitations, postural and gait abilities, and spatiotemporal gait features [30].

Treatment

RT is an important aspect of motor function that tends to decline with age. Slower response times are common in older persons, compromising balance and increasing the risk of falling. Both PBT and step training are intended to enhance their response speed and balance. Controlled disturbances are used in PBT to test sensory and motor systems. It is possible to do so using equipment such as balancing platforms, foam pads, or virtual reality systems. During training, older persons are subjected to controlled perturbations such as pushes and pulls while attempting to maintain balance. This helps with response time, muscular coordination, and overall stability. Participants improve their ability to adapt to unanticipated balancing issues over time [31] or therapist-applied perturbations [32].

Stepping skills can be improved by step training, which works by improving the capacity to take regulated steps in response to diverse stimuli. This type of exercise is essential for improving balance and reducing the chance of crippling falls, especially in older people. Step training's versatility, which provides a wide range of exercises according to the demands of the person, makes it particularly advantageous. These actions might be as simple as walking over barriers or as complex as reestablishing balance after interruptions or disruptions. Along with step training, PBT is a remarkable method for improving response time and strengthening balance in older people. These training techniques must be tailored to each individual's specific traits in order to be effective. When developing and carrying out these interventions, careful consideration must be given to individual variations in physical fitness, starting ability, and pre-existing medical issues. In short, a one-size-fits-all strategy should be eschewed in favor of customizing the training program to each person's unique requirements. It is essential to get the help of medical professionals or physical therapists before beginning step training or PBT. In determining a person's requirements and abilities, creating a tailored training plan, and ensuring that the training process is both efficient and secure, their expertise and experience are priceless. Working with these experts will help you maximize the advantages of step training. Engaging the skills of medical professionals or physical therapists is essential before beginning step training or PBT. They can analyze a person's requirements and talents, create a custom training plan, and make sure the training process is both efficient and secure thanks to their expertise and experience. Working together with these experts may maximize the advantages of PBT and step training, leading to a life that is safer and more well-balanced for persons who are trying to preserve or restore their physical equilibrium.

Age-related changes in response time are often treated by addressing cognitive abilities, physical health, and lifestyle variables that might affect RT. Due to changes in the brain and body with aging, response times might naturally go down. However, there are several methods and treatments that might lessen this deterioration and enhance older persons' RTs [29]. Table 1 mentions various studies regarding PBT and step training.

**Table 1:** Various studies regarding perturbation-based balance training and step training PBT: Perturbation-based balance training; COM: center of mass

Author and year	Design	Sample size	Intervention and outcome measure	Conclusion
Okubo et al. (2017) [33]	A meta-analysis and a systematic review	660 participants with risk of fall	Subgroup studies stratified by reactive and volitional stepping treatments demonstrated comparable efficacy in terms of fall rate and proportion of fallers.	Both reactive and volitional stepping therapies reduce falls among older persons by around 50%, according to the data.
Nørgaard et al. (2023) [34]	Randomized clinical trial	140 participants, functional elderly people	Participants in the intervention group were subjected to four 20-minute sessions of PBT, which included 40 slip, trip or combined slip and trip perturbations, as well as treadmill walking at their selected tempo.	In this experiment, individuals who received an 80-minute PBT intervention had a statistically insignificant 22% decrease in daily-life fall rates.
Lesinski et al. (2015) [35]	A meta-analysis and systematic review	375 healthy 65-year-old community-dwelling people who tested positive for at least one behavioral balance performance result	A random-effects model was used to compute the intervention-induced changes in balancing performance, which were then assessed for an overall intervention impact compared to passive controls.	In healthy older individuals, balance training improves proxies of static/dynamic steady-state, proactive, and reactive balance, as well as performance in balance test batteries.
Martelli et al. (2021) [36]	A pilot study	28 healthy community-living older adults	a battery of functional tests, and were evaluated for baseline cognitive performance using the Symbol Digit Modalities Test and the Trail Making Test	The study is the first to show that systematic variations in gait cause acute abnormalities in cognitive performance.
Pai et al. (1998) [37]	Randomized clinical trial	49 participants included 13 young, 18 older non-fallers, and 18 fallers	critical center of mass (COM) displacement-velocity values provides more accurate predictions for the initiation of protective stepping than a static model	The elderly, in general, and older fallers in particular, would commence steps at lower levels of disturbance severity than younger individuals.
Shen et al. (2012) [30]	Randomized clinical trial	28 patients with Parkinson's disease	For four weeks, perform repeated step training with visual signals and lower limb strength training. Limits of stability tests, postural and gait sub-scores were among the outcome measures used.	Repetitive step training with preceding signals can improve the limits of stability, postural and gait abilities, and spatiotemporal gait features in Parkinson's disease patients who have not fallen in the past 12 months.
Gerards et al. (2023) [38]	Randomized clinical trial	82 participants with a median age of 73 years	During standing and walking on the Computer Assisted Rehabilitation, unilateral treadmill belt accelerations and decelerations, as well as platform perturbations, were used. The environment and balance control were evaluated at baseline and one week after the intervention.	In community-dwelling older persons with a recent history of falls, participation in a PBT program with diverse perturbation kinds and directions had no different impact than standard care on clinical measures of balance control or fear of falling.

## Conclusions

This review concluded that the use of PBT and step training was effective in reducing RT in older adults, which decreased instability and increased lower-limb balance and strength, thereby improving their quality of life. This technique will lower the chance of falling and improve limb stability. It also implies the necessity for further literature on the subject. Specific brain structures must be activated in order for compensatory reactions to be produced in response to environmental perturbations. This implies that training balance recovery reactions ought to demonstrate acute training impacts on cognition.
